# A focusing method on refraction topography measurement

**DOI:** 10.1038/s41598-023-42950-0

**Published:** 2023-09-30

**Authors:** Huang Yequan, Guo Jingyun, Guo Yu, Cui Yan, Li Zhechuang, Dong Xuechuan, Ning Xiaolin

**Affiliations:** 1 School of Instrumentation Science and Opto-Electronics Engineering, Beihang University, Beijing, China; 2 Shenzhen Thondar Technology Co. Ltd, Shenzhen, China

**Keywords:** Applied optics, Refractive errors, Biomedical engineering

## Abstract

This paper introduces a novel focusing method Refraction Topography (RT) for wide-angle refraction measurement. The agreement of the test results obtained using RT is evaluated against simulation results and expected refraction. RT develops a refraction algorithm on fundus images at various focusing statuses. Unlike conventional techniques for peripheral refraction measurement, RT requires the subject to stare at a stationary fixation target. The refraction algorithm calculates the focus measure for multiple images at the Point of Interest and formulates them into a focus profile. The maximum focus measure correlates with the optimal focus position. Refraction Characterization Function (RCF) is proposed to translate the focus position into refraction determination, thus forming the refraction topography. The refraction characterization of RT optical system is performed using Isabel schematic eye. Three test eyes of − 15 D, 0 D, and + 15 D are defined, and expected refraction is obtained through simulation on an independent test schematic eye. Both simulation results and experimental results are obtained by combining the test eyes and RT system. Test results are compared with simulation results and expected refraction. The study demonstrates agreement among the test results, simulation results, and expected refraction on three test eyes.

## Introduction

Peripheral refraction has been a subject of study for several decades, and versatile measurement tools have been employed for different purposes. In the 1930s Ferree et al.^1–3^ pioneered the measurement of peripheral refraction by modifying a Zeiss parallax refractometer and mounting it onto a rotatable carriage enabling measurements along the horizontal meridian from temporal 60° to nasal 60°. In 1971, Hoogerheide and Rempt^4,5^ investigated the relationship between myopia and peripheral refraction in pilots using a retinoscope. This involves framing positive and negative lenses on a rotatable disc, which is placed in front of each eye of the subject. The examiner selected the appropriate lens based on the motion of the retinoscopic shadow until a neutral state was achieved. In 1977, Jennings and Charman^6^ introduced a double-pass photo-electric ophthalmoscopic method to objectively study the variation in image quality across the retina by analyzing the reflected image of a fine line, known as the line spread-function. In 1998, Navarro et al. utilized a laser raytracing method^7^ to investigate the monochromatic aberrations of the human eye along the temporal meridian. This method involved directing a narrow laser beam into the eye through a specific point on the pupil and capturing the aerial image of the retinal spot with a CCD camera. Peripheral refraction measurement with Shack Hartman aberrometer were performed by David^8^ in 2007 and Linda^9^ in 2009. In recent studies, the open-field refractor is commonly used by researchers, such as the SRW5000^10^ /SRW5001^11^ (Ajinomoto, Japan), NVision K-5001^12^ (Shin-Nippon, Japan) and WAM-5500^13^ (Grand-Seiko, Tokyo, Japan).

The instruments mentioned above share common characteristics as follows: (1) Each measurement focuses on a single Point of Interest (POI), which is controlled by fixation targets placed in multiple locations. (2) Subjects are required to actively adjust the viewing angles to move the POI, but there is no effective measure to ensure that they are effectively fixating on the target, nor to guarantee consistent head positioning on the head rest. (3) The process of measuring multiple points is time-consuming. For instance, in the pilot research conducted by Hoogerheide^4,5^, approximately 10 min were required to measure seven discrete points of the retina along the horizontal meridian for each eye.

In contrast to the approach of switching the fixation targets at different positions, Tabernero et al. introduced a fast-scanning photoretinoscope in 2009 by adjusting the angle of light entering the eye^14^. A full scan of 90° in one meridian took around 22 second^15^. In 2011, Bart implemented a Shack Hartman wavefront sensor and a light source onto a horizontally rotating swing^16^. More recently, in 2020, Zhenghua devised a method for two-dimensional peripheral refraction measurement by establishing evenly spaced vertically aligned fixation targets^17^. Each fixation target facilitated peripheral refraction measurement along a single meridian and measurements from multiple meridians were combined into two-dimensional representations using image processing techniques.

Spontaneous eye movements^18^ and fluctuations in aberrations^19^ have been observed during focused attention occurring at different frequencies. Therefore a faster measurement is always favorable for overcoming the dynamic variations in the eye’s optical system^14^.

To address this need for faster measurements, a focusing-based method called Multispectral Refraction Topography (MRT) was proposed (Thondar, China)^20–22^. MRT was first introduced based on an optical system of Multispectral Fundus Camera. The MRT function utilizes one wavelength of 850 nm rather than multispectral wavelengths, so Refraction Topography (RT) is called in this paper. RT enables the simultaneous measurement of refraction for spatially distributed points. The method features a single fixation target and captures dozens of fundus images allowing for the measurement of millions of points in seconds. In this study, the principle of MRT is detailed, the optical system is upgraded with a telecentric imaging module, and a peripheral refraction experiment is performed on three test eyes of − 15 D, 0 D, and + 15 D. The test results are evaluated against simulation results and expected refraction.

## Methodology

The methodology employed in this study uses an optical system of Multispectral Fundus Camera (Thondar, China) with focus optics^23^. This camera is capable of capturing a series of images at different focus positions using infrared wavelength. A focusing-based method is applied to determine the optimal focus position, and Refraction Characterization Function (RCF) is proposed to translate the focus position into refraction determination, thus forming the refraction topography.

### Optical system

**Figure 1:**
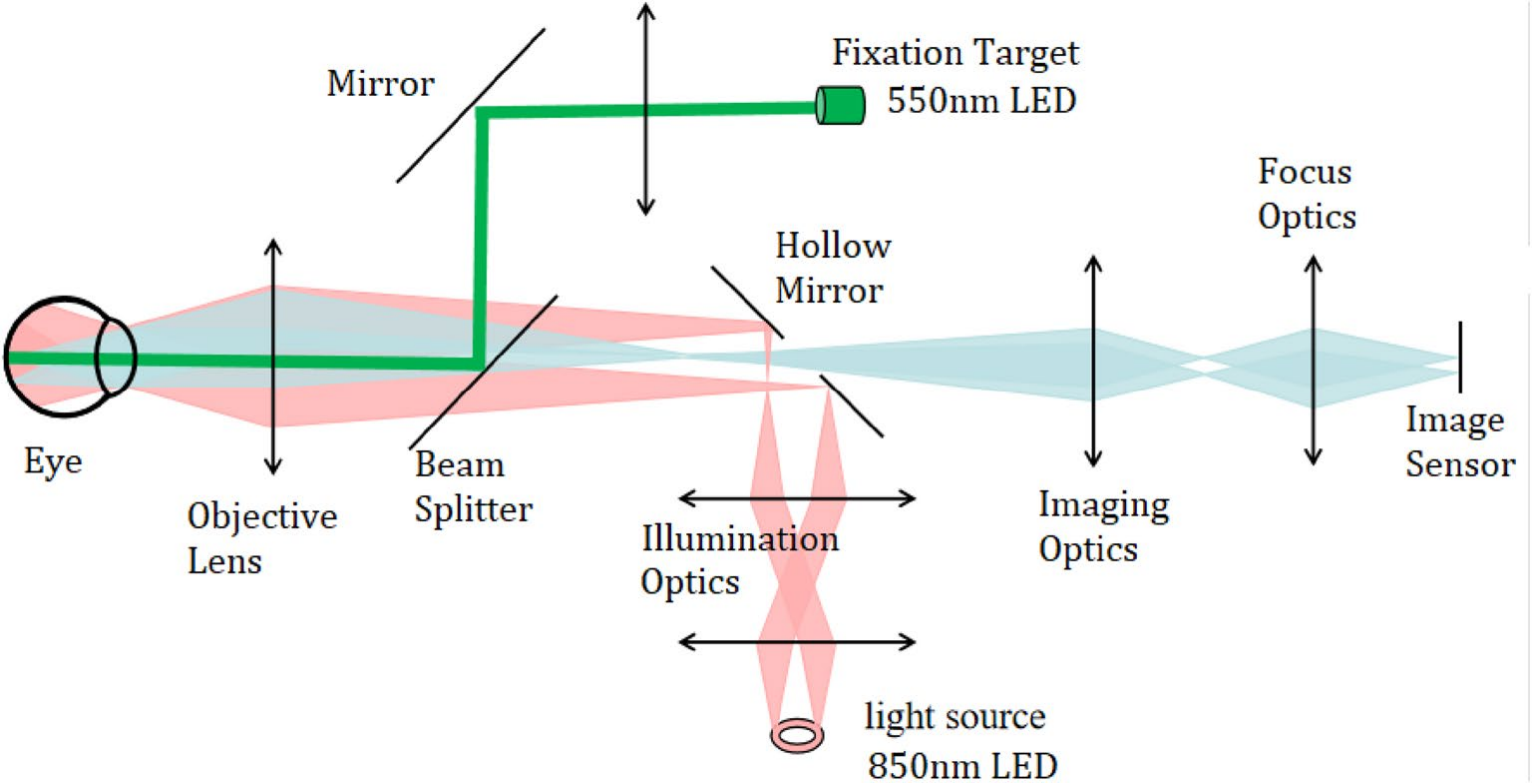
Optical system diagram for refraction tomography.

The optical system used in this study consists of three main components: illumination module, imaging module and fixation target module^24^. Figure 1 provides a diagram illustrating the optical system. The illumination module adopts an 850 nm near-infrared light source along with illumination optics to project evenly distributed illumination onto the fundus. To prevent stray light affecting the measurement, a ring-shape light source and a hollow mirror are employed to restrict the stray light produced by the corneal reflection. In the imaging module, the light reflected from the retina passes through the central hole of hollow mirror proceeds through imaging and focus optics, and finally reaches the image sensor. The telecentric optics are used in the imaging module. The fixation target of 550 nm LED guides the eye gaze and controls eye spontaneous movement.

The field of view of the system is set to 60°, which is equivalent to an eccentricity of 30°. The entrance pupil size is 1.4 mm in diameter, which is smaller than the typical diameter of human pupil ranging from 2.5 to 4.0 mm. The effective digital resolution of the system is 1024 × 1024 pixels, with a pixel size of 9.6 um. The amplification ratio from the retina to the image sensor is 2:1.

### Focusing model

**Figure 2:**
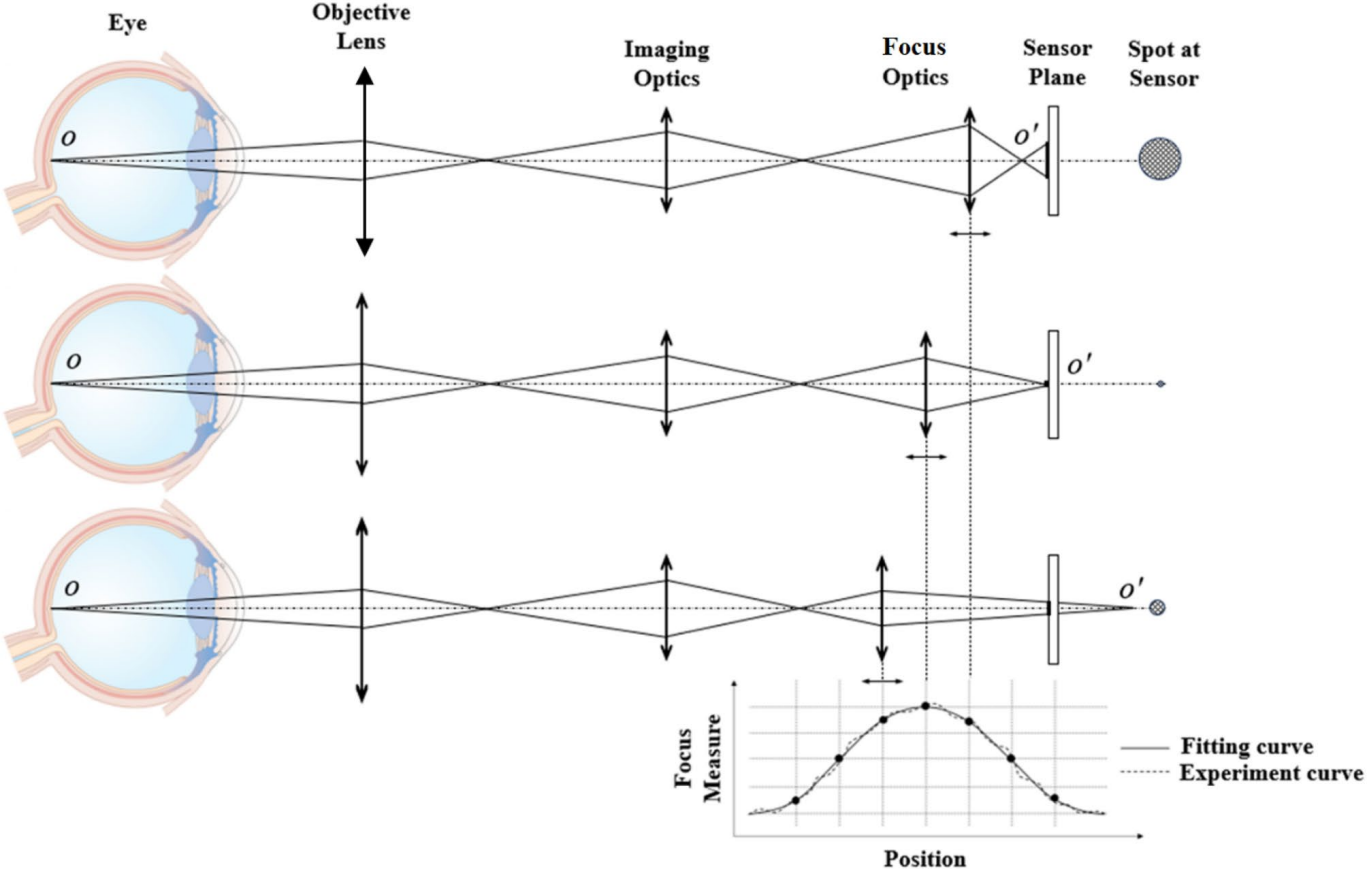
Focusing schematic of the RT.

Figure 2 depicts the focusing schematic of the RT system. In all three sub-figures, the eyes are identical and placed at the working distance of the RT system. The light radiates from object point O on the retina, passes through the eye’s refractive media, the optical system, and ultimately focuses a conjugate point O’ in image space. An image is formed on the sensor plane. In the three sub-figures of Fig. 2, the focus optics moves from right to left. According to Gauss's law, point O′ moves from left to right, and the size of the spot on the sensor varies accordingly. When point O′ coincides with the sensor plane, the best focusing is obtained, represented as the Minimum Confusion Disk (MCD). Consequently, the spot size on the sensor can be adopted as a focus measure effectively describing the distance between conjugate point O′ and the sensor plane^25^.

### Optimal focus position from focus measure

**Figure 3:**
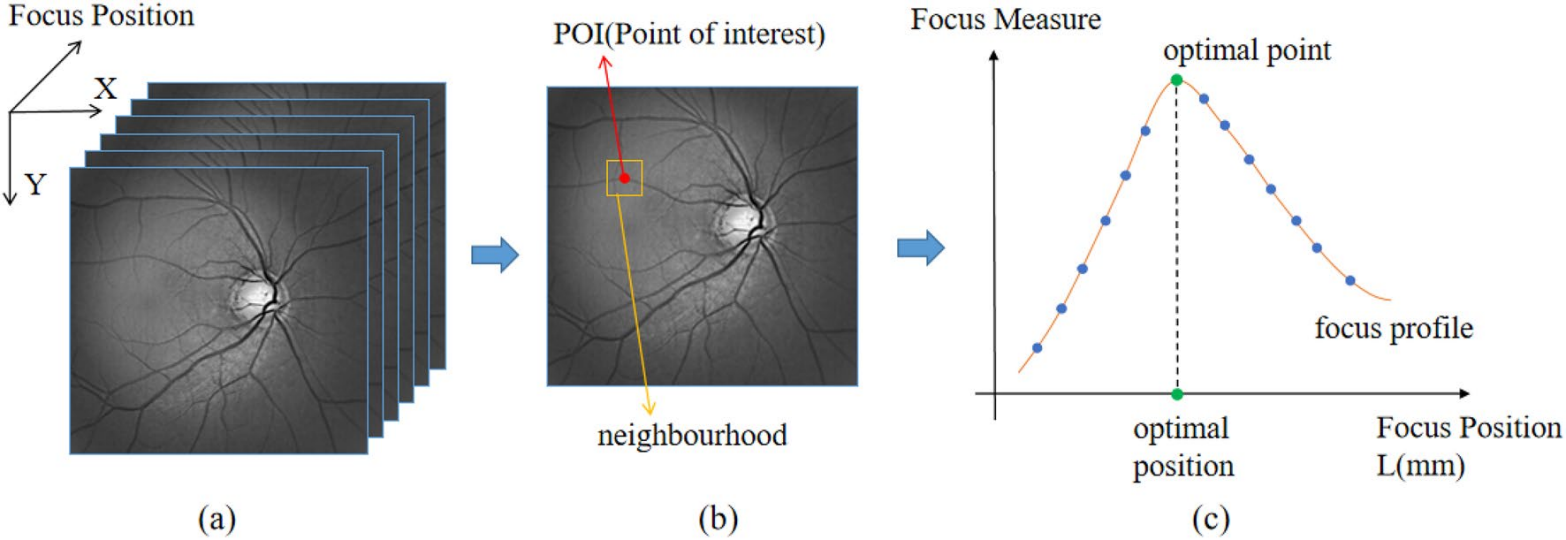
Determination of optimal position from image stack.

In the imaging process of the fundus camera, the object being captured is the retina surface rather than a single point. Thus, image spots of numerous points overlap on the sensor plane. The coordinates of a specific image point on the sensor remain constant despite changing the system's focal length due to the telecentric optics. The sharpness of captured fundus images varies (Fig. 3a). As a result, a focus measure based on a sharpness operator is performed for a POI (Fig. 3b) in the image^25–27^. In the paper, the Laplacian operator is employed as the focus measure, as it has been proved to be sound, monotonic and unimodal^25^.

The image and focus position serial is denoted as {(I_n_ (x, y), L_n_) | n = 1, 2, …, N}, where L_n_ indicates the n^th^ position of focus optics in fundus camera, and I_n_ is the corresponding image, N denotes the number of focus positions, and (x, y) is the Cartesian coordinates of pixel. For each image I_n_, a Laplacian image is obtained by convolving the image I_n_ with the Laplacian mask. Then, the focus measure at point (x, y) computed as the neighborhood average of Laplacian values:

1 $$ {\text{S}}_{{\text{n}}} \left( {{\text{x}},{\text{y}}} \right) = \frac{1}{{W^{2} }}\mathop \sum \limits_{i = x - W}^{x + W} \mathop \sum \limits_{j = y - W}^{y + W} \Delta I_{n} \left( {i, j} \right) $$

where ΔI_n_(i, j) indicates the Laplacian value of image I_n_ at pixel (i, j). The parameter W determines the neighborhood window size used to compute the focus measure. Neighborhood averaging is applied to help suppress noise and improve the estimation reliability of the focus measure.

For each POI, the focus measure is calculated on each image, resulting in a set of blue points as depicted in Fig. 3c. The blue points of all images collectively form a focus profile represented by red curve. The optimal point on the focus curve corresponds to the pixel with the highest S_n_ (x, y) value, indicating optimal focus position. The best focus at location (x, y) is achieved at this optimal focus position.

Outliers can occasionally arise due to factors such as blinking, mirror reflections, or weak illumination during capture of human eye image capture. To mitigate the impact of outliers, data fitting techniques are employed.

The Cartesian pixel coordinate (x, y) in image space can be uniquely interpreted as the Polar coordinate ($\uprho ,\uptheta$) in object space of the fundus, as shown in formula (2),

2 $$ \left\{ \begin{aligned} &\rho = k \cdot V \cdot M \cdot \sqrt {(x - x_{0} )^{2} + (y - y_{0} )^{2} } \hfill \\ & \theta = \arctan (\left( {y - y_{0} } \right)/\left( {x - x_{0} } \right)) \hfill \\ \end{aligned} \right. $$

where, (x_0_, y_0_) is the image center, k is the ratio from degree of field view to length on fundus, V is the width of sensor pixel, M is the ratio from the retina to the image sensor, $\uprho$ is an eccentricity measured in degree, and $\uptheta$ is a meridian measured in radians ranging from 0 to 2π.

### Refraction determination

The refraction determination process involves identifying the optimal point with the maximum focus measure in the focus profile. The refraction value can be computed based on the position of the optima, denoted as $\widetilde{\mathrm{L}}$.

**Figure 4:**
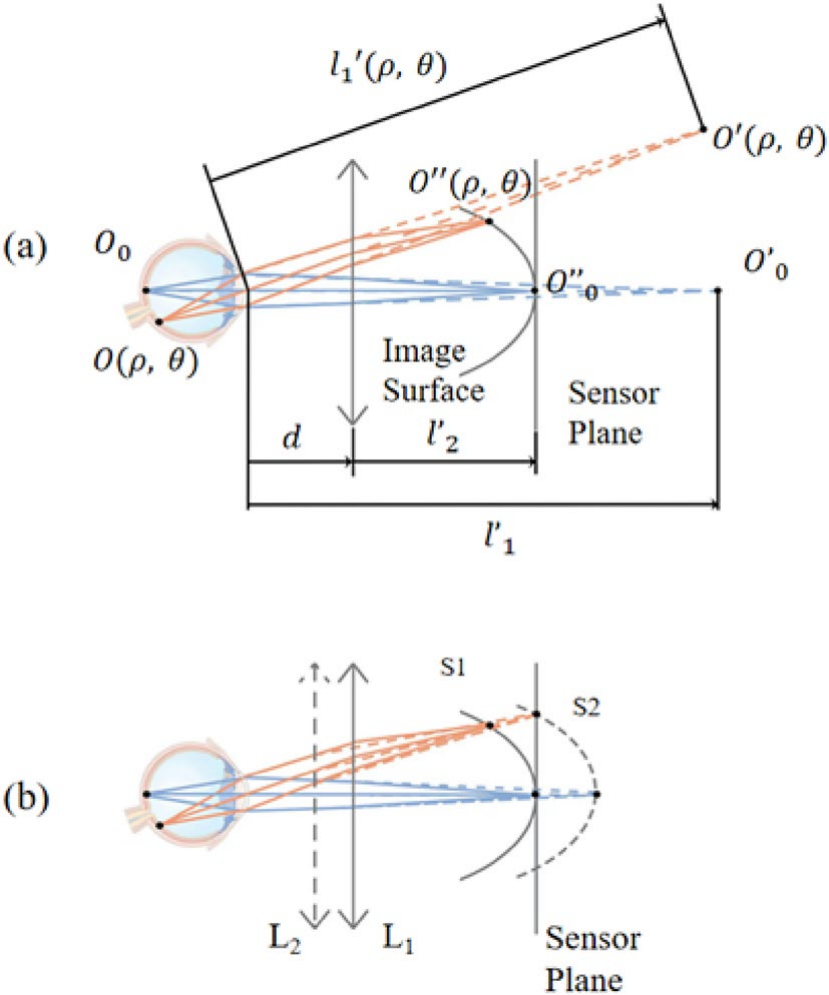
The positional relationship of object and image.

Let $\emptyset\left(\uprho ,\uptheta \right)$ represent the refraction of the observed eye at specific location ($\uprho ,\uptheta$). As shown in Fig. 4a, light originating from $\mathrm{O}(\uprho ,\uptheta )$ propagates through the eye’s refractive media and forms MCD at $\mathrm{O^{\prime}}(\uprho ,\uptheta$). The distance between the eye and the image point $\mathrm{O^{\prime}}(\uprho ,\uptheta$) is ${\mathrm{l}}_{1}^{\prime}(\uprho ,\uptheta$). According to the refraction definition, we have ${\mathrm{l}}_{1}^{\prime}(\uprho ,\uptheta )=-1/ \emptyset\left(\uprho ,\uptheta \right)$. The light continues to transmit through RT optical system, resulting in the formation of the secondary image spot $\mathrm{O^{\prime\prime}}(\uprho ,\uptheta )$. ${\mathrm{O}}_{0}$, ${\mathrm{O^{\prime}}}_{0}$ and ${\mathrm{O^{\prime\prime}}}_{0}$ are object point, image point, and secondary image point, respectively, located on the optical axis. The refraction determination is given in both of on-axis and off-axis situations.

#### On-axis situation

The positional relationship between ${\mathrm{O^{\prime}}}_{0}$ and ${\mathrm{O^{\prime\prime}}}_{0}$ follows the Gaussian formula,

3 $$\frac{1}{{\mathrm{l}}_{1}^{\prime}-\mathrm{d}}+\frac{1}{{\mathrm{f}}^{\prime}}=\frac{1}{{\mathrm{l}}_{2}^{\prime}}$$

where, $\mathrm{d}$ is the distance between the entrance pupil and the principal plane of the imaging module, ${\mathrm{l}}_{1}^{\prime}-\mathrm{d}$ and ${\mathrm{f}}^{\prime}$ are the object length and the focal length of the imaging module, and ${\mathrm{l}}_{2}^{\prime}$ is the image length of ${\mathrm{O}}_{0}^{\prime\prime}$. The detection optics are in the air, and the refractive index is assumed to be 1. ${\emptyset}_{0}$ is refraction on the axis. By substituting ${\mathrm{l}}_{1}^{\prime}=-1/{\emptyset}_{0}$ into formula (3), we obtain:

4 $$ \emptyset_{0} = \frac{1}{{\frac{1}{{\frac{1}{{{\text{l}}_{2}{\prime} }} - \frac{1}{{{\text{f}^{\prime}}}}}} - {\text{d}}}} $$

The RT system achieves focus by moving the position of the focus optics in imaging module, which causes a change in the focal length ${\mathrm{f}}^{\prime}$ of the imaging module and the image length ${\mathrm{l}}_{2}^{\prime}$. When an MCD is obtained on the sensor. The corresponding position of focus optics is defined as the focus position ${\tilde{\text{L}}}$. Given the optical design of the imaging module, ${\mathrm{f}}^{\prime}$, ${\mathrm{l}}_{2}^{\prime}$ and $\mathrm{d}$ are obtained with the focus position $\widetilde{\mathrm{L}}$. Consequently, ${\emptyset}_{0}$ is the function of the focus position $\widetilde{\mathrm{L}}$ as ${\emptyset}_{0}=f(\widetilde{\mathrm{L}})$.

#### Off-axis situation

As shown in Fig. 4b, assuming that the refraction of the eye is consistent across a wide angle, the conjugate surface S1 is associated with focus position L1. The vertex of S1 lies on the sensor plane but the off-axis image point lies on the left of the sensor plane. The peripheral displacement is contributed by aberrations like field curvature in the eye and the imaging module. By adjusting focus position to L2, the conjugate image surface S2 is obtained, and the off-axis image point moves onto the sensor plane. For the same refraction across the angle of the eye, the on-axis and off-axis result in conjugate image points on the sensor plane with different focus positions. As the RT optical system is rotationally symmetric, the field curvature $\mathrm{X}\left(\uprho ,\mathrm{ L}\right)$ in diopters is determined based on given eccentricity of $\uprho$ and the focus position of $\mathrm{L}$. The relationship between refraction and eccentricity is given by:

5 $$\emptyset\left(\uprho \right)= {\emptyset}_{0}(\mathrm{L})+\mathrm{X}\left(\uprho ,\mathrm{ L}\right)$$

Formulas (4) and (5) illustrate the intrinsic relationship between focus position, eccentricity, and refraction. Therefore, $\emptyset\left(\uprho \right)$ is defined as Refraction Characterization Function (RCF) as below:

6 $$\emptyset\left(\uprho \right)=\mathrm{ RCF}\left(\uprho ,\mathrm{ L}\right)$$

In measurement process, when a series of images are captured, one POI is selected at image coordinates (x, y) and the optimal focus position L is obtained according to above-mentioned method. The POI coordinates (x, y) can convert to Polar coordinates ($\uprho ,\uptheta$) according to Eq. (2). The refraction of the POI is calculated with L and $\uprho$ following Eq. (6).

The refraction determination contains two processes. The first process is refraction characterization based on combination of the RT imaging module and Isabel eye. The second process is the refraction determination with the focus position L. When L is obtained in the simulation system of the RT imaging module and test eye, the refraction determination is called “simulation result”. When L is from the test system of the physical RT system and the physical test eye, the refraction determination is called “test result”.

The refraction characterization is performed by ZEMAX ray tracing as follows:

1. Find the mapping between focus position L and vitreous thickness based on Isabel eye^28^.
  - Locate the Isabel eye at the working distance of RT imaging module in a coaxial manner.
  - Set the focus at sampled focus positions ${\text{L}}_{{\text{s}}} \left( {{\text{s}} = 1, \ldots ,{\text{S}}} \right)$, where S is the sample size.
  - Select a point on the fundus surface with eccentricity ${\uprho }_{{\text{t}}} \left( {{\text{t}} = 1, \ldots ,{\text{T}}} \right)$, where T is the sample size.
  - Perform ZEMAX simulation ($\uplambda =850\,\mathrm{ nm}$):
    i. Select an object point on the retina. Set the vitreous thickness as a variable^26^.
    ii Optimize the thickness above, until all the rays from the point focus to form the MCD on the image sensor of the imaging module.
    iii. Record the optimized vitreous thickness r_st_.
2. Simulate the refraction of Isabel eye ($\uplambda =543\,\mathrm{ nm}$) with vitreous thickness ${\mathrm{r}}_{\mathrm{st}}$ in ZEMAX
  - Select an object point in the air. Adjust the location of the object point in the air until the MCD is formed on the retina of Isabel eye (with the vitreous thickness ${\mathrm{r}}_{\mathrm{st}}$ and eccentricity ${\uprho }_{\mathrm{t}}$).
  - Record the object length ${\mathrm{l}}_{\mathrm{st}}$, and calculate the refraction ${\mathrm{R}}_{\mathrm{st}}=-1/{\mathrm{l}}_{\mathrm{st}}$.
3. Repeat steps (1) to (2) for other samples to obtain characterization matrix of ${\text{R}}_{{{\text{st}}}} \left( {{\text{s}} = 1, \ldots ,{\text{S}};{\text{t}} = 1, \ldots ,{\text{T}}} \right)$.
4. Select an appropriate model function with independent variables of focus position $\mathrm{L}$ and eccentricity $\uprho$, and perform data fitting with characterization matrix. We obtain the $\mathrm{RCF}\left(\uprho ,\mathrm{ L}\right).$

Note: Parameters in ZEMAX ray tracing are:

- Optimization function and reference: RMS, Spot Radius, Centroid
- Pupil integration method: Gaussian quadrate
- Ray number: 12 rings, 12 arms, 0 obscuration
- Ray aiming: real, robust slow

Three test eyes are defined to verify the accuracy of refraction determination by RCF with Isabel eye. Expected refraction is obtained through ray tracing by moving the object until achieving the minimum RMS spot on retina of test eyes in ZEMAX. The Expected refraction is given at various eccentricities. Three test schematic eyes are designed with central diopter of − 15 D, 0 D and + 15 D based on MCD criterion in Zemax. They are named − 15 D test eye (ID: M1), 0 D test eye (ID: M2), and + 15 D test eye (ID: M3), respectively. The parameters for test schematic eyes are presented in Table 1.

**Table 1:** Parameter specification of test schematic eyes.

Material	H-K10
Radius of the anterior surface (cornea)	8 mm
Radius of the posterior surface (retina)	− 12.244-mm (M1) − 10.252 mm (M2) 8.668 mm (M3)
Posterior surface processing	Grinding
Posterior surface roughness	Sa = 0.608 um
Total length	30.19 mm (M1) 23.32 mm (M2) 18.99 mm (M3)
Field of view	60°
Diameter of pupil	3 mm
Pupil distance from anterior surface	3.6 mm
Benchmark wavelength	546 nm

Both simulation result and test result of three test eyes are calculated with RCF in this paper.

## Experiments and results

In this section, system characterization is conducted using the Isabel eye as a basis^28^. Expected result and simulation result of three test eyes are obtained by ZEMAX ray tracing. Furthermore, the test result of test eyes is observed. Finally, both simulation result and test results are compared with the expected refraction at various eccentricities.

The measuring range for refraction is from − 15 D to + 15 D with a maximum scanning range of − 20 D to + 20 D. The focus position interval between two adjacent image captures is 0.19 mm, corresponding to a mapping of 0.5 D in refraction. This generates a series of 81 fundus images in frame rate of 30fps. The focus measure adopts the Laplacian sharpness operator with a window size of 5 pixels and the neighborhood averaging window size is 25 × 25 pixels. A fourth-degree polynomial is applied to fit the focus profile.

### Experiments

#### RCF modeling

To obtain the refraction characteristic function $\mathrm{RCF}\left(\uprho ,\mathrm{ L}\right)$, a total of nine focus positions and seven eccentricities were sampled to simulate the refraction based on the Isabel schematic eye^28^. The sampled eccentricities are 0°, 5°, 10°, 15°, 20°, 25°, 30°. The sampled focus positions are 7.728, 5.796, 3.864, 1.932, 0, − 1.932, − 3.864, − 5.796, and − 7.728 mm. The refraction at each sampled point was simulated at the point with sampled eccentricity and focus position using ZEMAX. Each point on the retina was set as object and the vitreous thickness was treated as a variable. The ray tracing was performed through the refractive media and the RT optical system to ensure that the MCD was formed on the sensor. Subsequently, the object was moved in the air back and forth until the image was formed at the given point on the retina. The refraction was calculated based on the optimized object length in the air.

**Figure 5:**
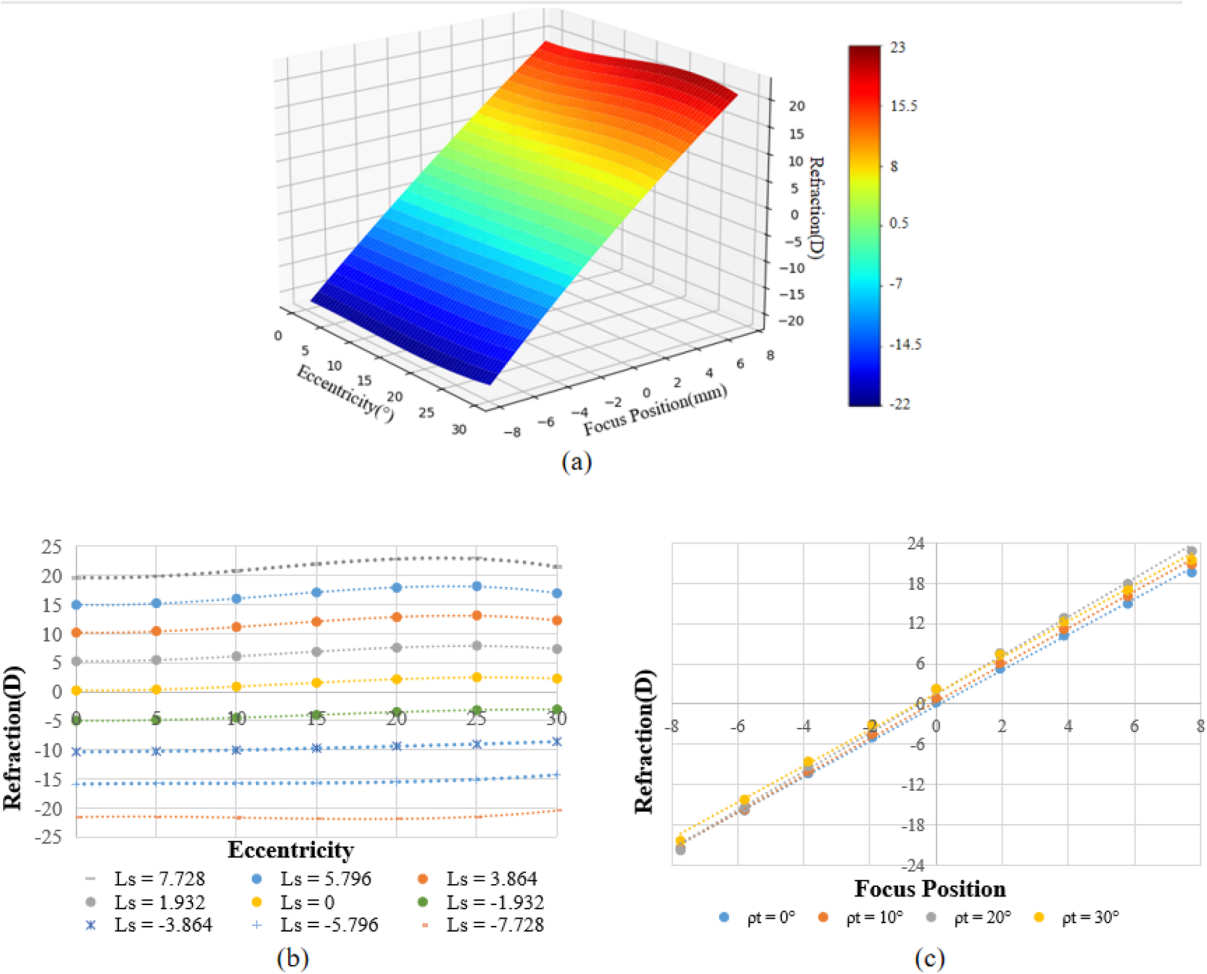
Relationships between the simulated refraction in Isabel eye with focus position and eccentricity. (**a**) The 3D model of RCF. (**b**) Relationships between the simulated refraction and eccentricity at various focus positions. (**c**) Relationships between the simulated refraction and focus position at various eccentricities.

By ZEMAX simulation, 63 sample points of (ρ, L, R) were obtained. A bivariate fourth-degree polynomial was applied on the 63 points to fit the simulated refraction data with respect to eccentricity and focus position, resulting in the formation of the $\mathrm{RCF}\left(\uprho ,\mathrm{ L}\right)$. The 3D model of the fitted RCF is shown in Fig. 5a. To evaluate the performance of the fitting RCF function, the residual error and goodness were calculated with the simulated refraction and fitted refraction of the sample points. The residual error of the fitting function was found to be + 0.055 D, and the goodness of fit was 0.996.

Figure 5b shows the relationship between the simulated refraction and eccentricity in Isabel eye at various given focus positions. The field curvature exists at all focus positions ranging from + 1.13 to + 2.08 D at an eccentricity of 30°. E.g. When the focus position Ls = − 7.728, the simulated refraction of Isabel eye are − 21.49 and − 20.36D at the eccentricity of 30° and 0° respectively. The field curvature is + 1.13D at this focus position. The field curvature increases as the focus position increases. Figure 5c shows the relationship between the simulated refraction and focus position at different given eccentricities. For all eccentricities, the refraction increases with the focus position increases. The central refraction range is (− 21.49 D, + 19.63 D), and the peripheral refraction range is (− 20.36 D, + 21.47 D) at an eccentricity of 30°, covering the measuring range of (− 15 D, + 15 D).

#### Simulation and measurement on test eye

In order to assess the performance of the system on the test eyes, the expected refraction is calculated by ZEMAX simulation. The object is moved back and forth until MCD is formed at the retina of the test eyes in the ZEMAX simulation, assuming a wavelength of 546 nm. The expected refraction R is calculated based on the object distance L, where R = 1/L. It is ensured that the deviation of the peripheral refraction from center refraction is controlled within 0.5 D across the wide-angle of view for all three test eyes.

The simulation result is obtained by integrating the test schematic eyes M1, M2, and M3 with the RT optical system. The focus position is adjusted in ZEMAX to achieve the optimal focus position where the point on the fundus and the image point on the sensor plane are conjugate. The RCF is applied to translate the focus position into refraction.

To obtain the measurement results on the test eye, the test schematic eyes are positioned at the working distance of the RT optical system. The test results are achieved through a physical experiment setup and the RCF application. The test schematic eyes are fabricated into physical test eyes and are equipped with an additional mechanical socket that connects them to the RT optical system at the appropriate working distance. The central refraction of RT optical system is calibrated with a standard test eye^29^ of + 1D (typically used for optometer calibration) by adjusting the sensor plane back and forth. However, the peripheral refraction is not calibrated in this experiment.

## Results

**Figure 6:**
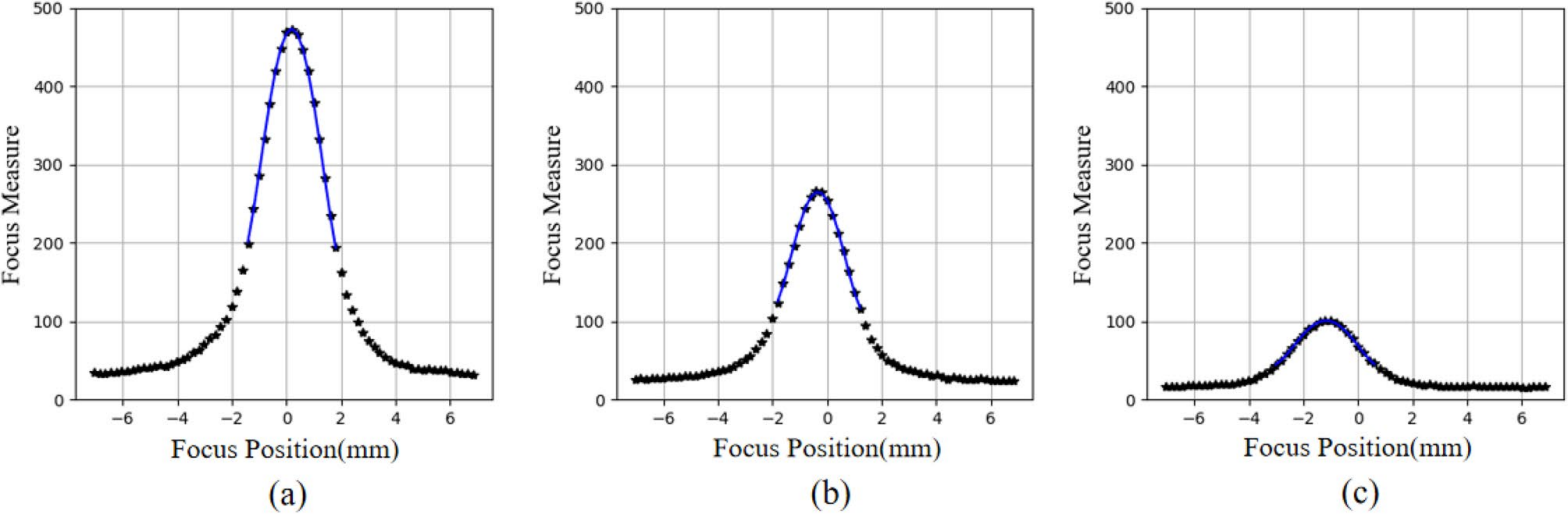
Focus profiles at different eccentricities. (**a**) Eccentricity of 0°. (**b**) Eccentricity of 15°. (**c**) Eccentricity of 30°.

Before the comparison, some interim deliverables on M2 measurement are provided to give a concrete understanding of the RT principle. In total, 81 images were captured. Figure 6 presents the focus measure profiles at eccentricities of 0°, 15°, and 30°. The R-squared values of the fits are 0.997, 0.991, and 0.99, respectively. The maximum focus measures are 485, 268 and 103. It the observed that as the eccentricity increases, both the R-squared values and the maximum focus measure decrease.

**Figure 7:**
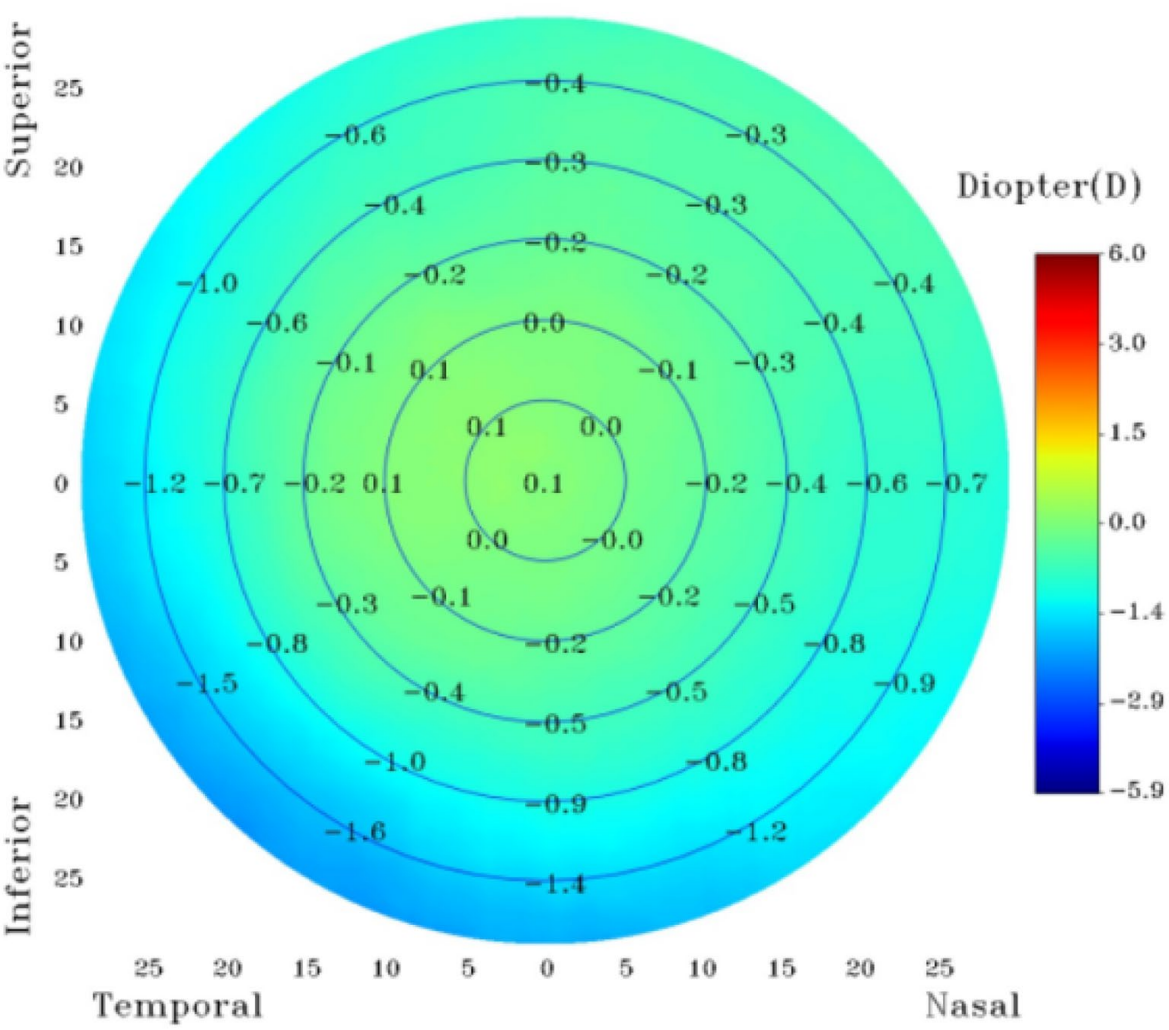
An example of the refraction topography for M2 Test Eye.

For each pixel, the optimal focus position is resolved, and the RCF presented in Fig. 5a is applied to convert focus position into refraction. The resulting pseudo-color refraction topography for the M2 test eye is depicted in Fig. 7, where the refraction values are arranged pixel by pixel. The FOV is 58°, slightly less than the intended 60°, due to additional size required for focus measure calculation. The refraction topography displays five concentric circles, each indicating a 10° FOV. The color scheme represents hyperopia (red) and myopia (blue), with the refraction decreasing outward in all meridians. The refraction distribution exhibits a myopia tilt towards the temporal-inferior side. The sums of refraction in two opposite meridians at the same eccentricity remains consistent. Consequently, a circular average is adopted as the test result for comparison with the simulation result below.

Table 2 shows the comparison of test results, simulation results and expected refraction for eyes M1, M2, and M3 at eccentricities ranging from 0° to 30° in 5° intervals. The simulation error represents the deviation between the simulation result and the expected refraction, reflecting the system error of the RT method. The simulation errors of all three eyes are controlled within ± 0.26 D with M2 exhibiting the highest simulation performance and a maximum error of 0.07 D. M1 has the highest simulation errors, especially at peripheral eccentricities. The errors are + 0.16 D (20°), + 0.23 D (25°), and − 0.26 D (30°). The central simulation errors for three test eyes are + 0.01D, − 0.03D, and + 0.01D respectively.

**Table 2:** Comparison of expected refraction, simulation results, RT test results and autorefractor test results for − 15D, 0D, + 15D test eyes at eccentricities from 0° to 30° in 5° interval.

Test eye	Eccentricity $\rho$ (°)	0	5	10	15	20	25	30
M1 − 15D	Expected refraction (D)	− 15.38	− 15.4	− 15.46	− 15.53	− 15.55	− 15.43	− 15.07
	Simulation result (D)	− 15.38	− 15.31	− 15.37	− 15.44	− 15.39	− 15.2	− 15.32
	Simulation error (D)	+ 0.01	+ 0.09	+ 0.09	+ 0.09	+ 0.16	+ 0.23	− 0.26
	RT Test result (D)	− 15.23	− 15.30	− 15.45	− 15.64	− 15.81	− 15.94	*
	RT Test error (D)	+ 0.15	+ 0.10	+ 0.01	− 0.11	− 0.26	− 0.51	*
M2 0D	Expected refraction (D)	− 0.31	− 0.33	− 0.36	− 0.4	− 0.42	− 0.35	− 0.11
	Simulation result (D)	− 0.33	− 0.33	− 0.31	− 0.35	− 0.37	− 0.32	− 0.18
	Simulation error (D)	− 0.03	− 0.01	+ 0.05	+ 0.05	+ 0.04	+ 0.03	− 0.07
	RT Test result (D)	+ 0.09	+ 0.10	− 0.02	− 0.23	− 0.53	− 0.81	*
	RT Test error (D)	+ 0.40	+ 0.43	+ 0.34	+ 0.17	− 0.11	− 0.46	*
M3 15D	Expected refraction (D)	+ 14.75	+ 14.74	+ 14.72	+ 14.7	+ 14.69	+ 14.72	+ 14.93
	Simulation result (D)	+ 14.75	+ 14.59	+ 14.68	+ 14.74	+ 14.74	+ 14.67	+ 14.91
	Simulation error (D)	+ 0.01	− 0.15	− 0.04	+ 0.04	+ 0.05	− 0.05	− 0.03
	RT Test result (D)	+ 15.24	+ 14.92	+ 14.90	+ 14.86	+ 14.80	+ 14.74	*
	RT Test error (D)	+ 0.49	+ 0.16	+ 0.18	+ 0.14	+ 0.11	+ 0.02	*

* Invalid results for FOV limitation, some extra size is required for focus measure calculation.

The test error is obtained by subtracting the test result from the expected refraction, encompassing the system error, fabrication error, and assembly error of a single physical RT system. The central test errors for M1, M2, and M3 are + 0.15 D, + 0.40 D, and + 0.49 D respectively. With the increasing of eccentricity, both the test result and test error exhibit a trend towards increased myopia. The residual field curvature at 25° is − 0.66 D, − 0.88 D, and − 0.45 D respectively.

## Discussion

The proposed method of Refraction Topography (RT) offers a novel approach to wide-angle refraction measurement using a fundus camera with wide-angle focusing capability. The RT focuses on the control logic adjustment of focus optics, refraction characterization, and algorithm design. It enables rapid and automatic acquisition of refraction in wide field of view.

Formula (3) and (4) illustrate the explicit relationship between refraction and parameters of optical system theoretically, providing guidance for the realization of refraction topography in three directions: (1) focal length adjustment, (2) sensor plane adjustment, and (3) both of focal length and sensor plane change. In this article, the focus optics in RT optical system follows the second approach. However, due to the complexity of the instrument with over 20 thick lenses in wide angles, it is impractical to define the refraction through precise formula deduction alone. To address this challenge, the RCF is proposed to translate the optical parameters into refraction. RCF serves three functions: (1) bridging measurement at 850 nm wavelength with the refraction at 546 nm wavelength, (2) compensating for the severe field curvature introduced by the optical system, and (3) correlating the 3 mm diameter of Isabel eye with the 1.4 mm entrance pupil size of the optical system. The specific form of the RCF depends on the design of the RT optical system, the working wavelength, and the diameter of entrance pupil. In this paper, a bivariate fourth-degree polynomial is selected as the model for refraction with eccentricity and focus position. Alternatively, other alternative fitting models could be chosen to strike a balance between the fitting accuracy and universality. Additionally, the sampling range and interval of eccentricity and focus position could be adjusted to improve the fitting performance.

The RCF is trained on the Isabel eye and applied in observed eye, which could be the test eye in this paper or human eyes in clinical measurement. The similarity between the Isabel eye and the observed eye will impact the measurement accuracy. The detailed design of the three test eyes has great deviation from the Isabel eye, which raise the worse situation for accuracy evaluation. The test eyes have less interfaces so that the reflection can be well controlled in the test and more feasible to fabricate. Although the aberration characteristics of the test eye differ from the Isabel eye. The simulation error in Table 2 was well controlled within ± 0.26 D. Notably, the maximum simulation error of only ± 0.07 D for 0 D test eye suggests that reducing aberration differences can lead to a further reduction in simulation error. It is reasonable to expect that the simulation error would be smaller between the Isabel eye and the human eye because of their closer aberration characteristics^28^. The well-controlled simulation error within the study demonstrates the rationality and validity of the RT methodology.

The RT system in this study was calibrated only for central refraction using a standard model eye, typically used in optometer calibration. The peripheral refraction was not calibrated in the experiment. As a result, the asymmetry observed in Fig. 7 can be attributed to fabrication errors and assembly errors in the RT optical system. While the test eye used in the experiment was rotationally symmetric, an azimuthal averaging method was performed to attenuate the tilt impact. However, some residual field curvature ranging from 0.66 D to 0.88 D at 25° remained even after applying the RCF. Further research is needed to develop a calibration method specific to individual RT optical systems in order to compensate for the tilt and residual field curvature. The RCF focuses on systematic compensation from a design perspective. Calibration, on the other hand, is concerned with the individual characteristics of each RT optical system. These test results at an eccentricity of 30° are deemed invalid due to the limited FOV of the test eye, which only provides a 60° FOV. To obtain accurate measurement at higher eccentricities, the FOV needs to be expanded, and additional test eyes with finer granularity are required to capture the refraction trends at various eccentricities.

The significant test error observed in the central refraction for M2 and M3, despite calibration using a + 1 D standard eye, can be attributed to several potential causes, including standard eye deviation, test eye deviation, and definition difference between expected refraction and standard eye. The definition of expected refraction relates to the location of MCD. The light bundle presents a distorted and chaotic pattern, especially in aberrated and highly refracted states. Different merit functions could result in different refraction values. Indeed, the Laplacian operator has been tested for focus without astigmatism (and certainly high-order aberrations). Apparently, for the levels of aberration present in this study, it performs reasonably well.

RT has nearly one million pixels covering continuous wide-angle of FOV. The interval of pixels is 0.06°, which is more intensive than two-dimensional refractor VPR with interval of 1°in one horizontal meridian^17^. The RT measurement takes 2.7 s in image capture. VPR takes 1.3 s for each horizontal meridian and manually adjust the distant fixation target to switch to another meridian^17^. Even 10 horizontal meridians would take several minutes. VPR can measure aberrations of various orders. RT measurement is limited to refraction.

## Conclusion

This focusing method of RT based on the telecentric imaging module has provided good agreement between simulation result and expected refraction, as well as a basic consistency between the test result and expected refraction on three test eyes (− 15 D, 0 D, + 15 D). This validates the concept of the RT methodology. However, to improve the accuracy and reliability of the RT system, further calibration is necessary to correct some residual field curvature and central refraction errors. Moreover, clinical studies are required to validate the repeatability, reproducibility, and accuracy of the RT system in real-life human eyes. Gathering experience data from a larger population can help refine and enhance the accuracy of the RT system.

While this paper presents an overview of the RT methodology, several engineering details can be further studied. For example, besides measuring the spherical equivalent refraction, calculations of other aberrations such as astigmatism could be explored based on one-dimensional focus measure. High-order peripheral aberrations can also be computed based on given series of images. Optimization of detailed optical parameters such as aperture stop size and position, as well as illumination, can contribute to improving measurement accuracy and reliability. Factors like focus position interval, range, and motor speed can influence the measurement range and time. Furthermore, the selection of focus measure operators, and pre-processing techniques are also areas of interest for future research and refinement of the RT methodology.

## Data Availability

The datasets generated and/or analyzed during the current study are not publicly available due [the data is provided by Thondar company, who does not permit to disclose the raw datasets] but are available from the corresponding author on reasonable request.
